# Influential Parameters of Starching Process on Mechanical Properties of Yarns Intended for Multifunctional Woven Fabrics for Thermal Protective Clothing

**DOI:** 10.3390/polym13010073

**Published:** 2020-12-27

**Authors:** Ivana Schwarz, Stana Kovačević, Ivana Vitlov

**Affiliations:** 1 Department of Textile Design and Management, Faculty of Textile Technology, University of Zagreb, Prilaz baruna Filipovića 28a, 10000 Zagreb, Croatia; stana.kovacevic@ttf.unizg.hr; 2 School of Applied Arts and Design, Perivoj Vladimira Nazora 3/3, 23000 Zadar, Croatia; ivana.vitlov@skole.hr

**Keywords:** starching, yarns for thermal protective clothing, mechanical properties, laboratory starching machine

## Abstract

The investigation of influential parameters of the starching process on mechanical properties of yarns intended for multifunctional woven fabrics for thermal protective clothing was performed on four different yarn samples starched on an innovative starching machine, adapted to industrial starching conditions. The starching was conducted with two different processes with different starch mass concentrations: the standard starching process and a newer starching process (with yarn prewetting). Based on the results obtained, it can be concluded that starching positively affects all the properties of tested samples and that the increase of starch mass concentration is not accompanied by the improvement of those yarn properties. Synthetic polymer fibers that achieve satisfactory yarn strength need to be starched with lower starch mass concentrations in order to retain the breaking properties and to be protected from abrasion and static electricity, which occurs during the weaving process. The yarn prewetting starching process shows significantly better results than the standard starching process, especially for aramid yarns, where abrasion resistance increased from 42 to 135%. Therefore, we can conclude that the goal of starching such yarns is aimed at increasing the wear resistance. Linear regressions and correlations between the values of breaking properties and abrasion resistance obtained by the testing and their values that were estimated by the analysis show a high correlation coefficient.

## 1. Introduction

In today’s wide field of protective textiles, great emphasis is on the production of woven fabrics, where design, construction solutions and use of new and diverse textile fibers can influence the achievement of a wide range of requirements, in order to obtain multifunctional multilayer woven fabrics [1,2].

Beside the ability to provide a high level of protection, protective clothing must have the properties of comfort and durability. The fabrics used for this purpose are mostly single-layer flat products, which represent the top layer (in composite materials) of protective clothing. Composite materials used for protective purposes are composed of several diverse materials. The upper layer is a woven fabric, which is joined by other layers that differ from woven fabric by their structures, raw materials and properties. It is becoming more demanding, complex and difficult to achieve certain material properties, prescribed by standards for a particular purpose, and it is even more difficult to achieve the sustainability during use. Therefore, finishing processes (surface treatments and layering) are conducted on the material, which simultaneously affect the property of breathability, resulting in the insufficient material comfort [3,4,5,6,7].

Woven fabrics are complex structures whose ultimate properties are conditioned by parameters at the macro-level, meso-level and micro-level. The macrolevel implies the woven fabric construction parameters, the meso-level implies the yarn structural parameters of which the fabric is composed, while the microlevel implies the properties of the fibers. Thus, fibers, with all their properties, carry the fundamental properties of any woven fabric [8].

Fibers used today for production of woven fabrics for protective clothing, which have a high degree of thermal protection, are aramid fibers, especially meta-aramid fibers. They are characterized by exceptional properties such as fire resistance, thermal stability, thermal conductivity, thermal resistance (does not lose strength properties under permanent load at elevated temperatures up to 180 °C), exceptional mechanical properties (high strength properties), good heat insulation, resistance to peeling, antistatic properties and are easy to maintain [9]. Very significant are modacrylic fibers (MAC), which are characterized by high flame retardancy, satisfactory abrasion resistance, softness and pliability, suitability for dyeing and durability. Blends of these fibers with polymer FR (flame retardancy) fibers are most often used for thermal protection, but their independent use is also important. In order to achieve the comfort, other fibers are also used in blends with these fibers, such as cotton and polyamide (PA) fibers, as the inner layer of protective clothing [10,11,12,13]. An increasing share of synthetic fibers in woven fabric production processes (especially technical fabrics for protective clothing), have relatively small surface masses, extremely fine threads and relatively high densities, so they require intensive and continuous monitoring during fabric production stages, in order to meet set standards. Some of the filament or spun synthetic yarns have satisfactory physical–mechanical properties, such as strength, abrasion resistance, elasticity, as well as resistance to long-term cyclic stresses, so they do not need to be starched. Since the starching process is the most expensive process in woven fabric production, it should be avoided whenever possible. However, sometimes its implementation is necessary in order to improve the yarn physical–mechanical parameters relevant to the further weaving process, in terms of: reducing the number of warp thread breaks to a minimum, achieving the maximum degree of weaving machine utilization and increasing the quality of the finished fabric. There are no standardized methods that will give the exact information on which yarn should or should not be starched. Therefore, a comprehensive analysis of yarn, target woven fabric, weaving machine and production process conditions is needed to get the best answer to the question: whether a particular yarn needs to be starched, to what extent or whether the starching is even necessary [14,15,16,17,18,19,20,21,22,23,24,25,26,27,28,29].

In this paper, the purpose of starching yarns intended for the production of multifunctional woven fabrics for thermal protective clothing will be investigated. The question arises, whether these yarns need to be starched, and if so, by which starching process and under what starching conditions. The implementation of the research and the comparative analysis of the results obtained will provide insight into the yarn mechanical properties, considering very important ecological and economic segment.

## 2. Materials and Methods

**Figure 1:**
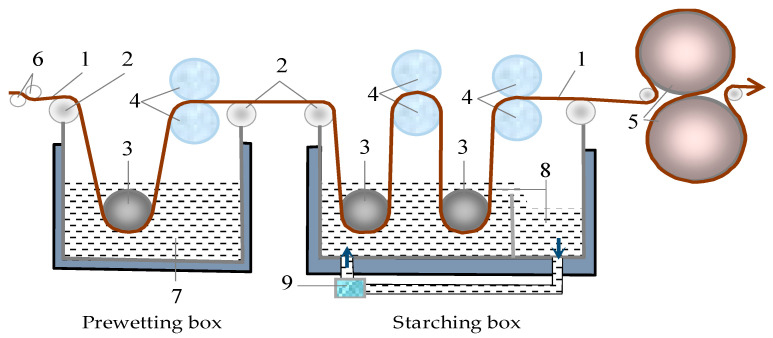
Scheme of a laboratory starching machine, where: 1—yarn, 2—rollers for guiding threads, 3—rollers for immersion in water and starch mass, 4—rollers for squeezing out excess water and starch mass, 5—rollers for contact drying, 6—tensiometer, 7—hot prewetting water, 8—starch mass, 9—starch mass circulation pump.

Due to easier and more economical regulation of starching conditions, a laboratory starching machine for samples preparation was used. Two starching processes were applied: the standard starching process and the newer prewetting starching process. The possibility of implementing both starching processes on a laboratory starching machine, constructed at the University of Zagreb, Faculty of Textile Technology, Zagreb, Croatia (recognized as consensual patents no. PK20070247 and PK20070248, State Intellectual Property Office of the Republic of Croatia) is significant. Laboratory starching conditions are adapted to industrial ones, and the method of starching is shown in Figure 1.

The tests necessary to draw relevant conclusions were performed on four different yarn samples whose basic properties are shown in Table 1.

These fibers are used specifically in conditions of thermal protection, where it is extremely important to know and consider their advantages and disadvantages (Table 2).

Starch agents and their share in the starch mass, which are commonly used in the industry for the types of yarns listed above, were selected for this research, because they are most suitable for the selected yarns (due to yarn types and fibers), as well as for the requirements of the starching process and further weaving process.

Starch agents used for starching processes were Fibrosint C75 (Pulcra Chemicals GmbH; synthetic polymer, Geretsried, Germany) and Inex 773C (Pulcra Chemicals GmbH; chemical composition: polyvinyl alcohol, Geretsried, Germany) in concentrations of 5%, 7% and 10%, and according to the proportions shown in the Table 3.

During the starching process, all conditions affecting the starch pickup were achieved and kept constant (Table 4). The process was carried out by simultaneous starching of five threads at a length of 200 m.

Determination of yarn mechanical properties was performed according to the following methods:

- Yarn breaking properties, which include the following properties: breaking force F (cN), elongation at break ε (%) and tenacity σ (cN/tex)—according to ISO 2062, on the Statimat M tensile tester, t.t. Textechno, Mönchengladbach, Germany;
- Yarn abrasion resistance, A (no. cycles)—the abrasion resistance test was performed on a Zweigle Abrasion Tester G551, Zweigle, Reutlingen, Germany, where each of the 20 threads, under a load of 20 g, is simultaneously subjected to the abrasion process until the thread breaks. The movement of the roller coated with sandpaper (finesses 600), left–right and rotating around its axis, achieves a certain intensity of yarn and sandpaper abrasion. During abrasion, the thread weakens and at the moment when the mass of the weight suspended on the thread exceeds the yarn strength, an interruption occurs and the number of movements is registered.

## 3. Results

The reliability of the results obtained by the conducted tests was confirmed and supported by statistical processing and analysis, where the indicators of result variability and reliability are shown in Table 5.

**Figure 2:**
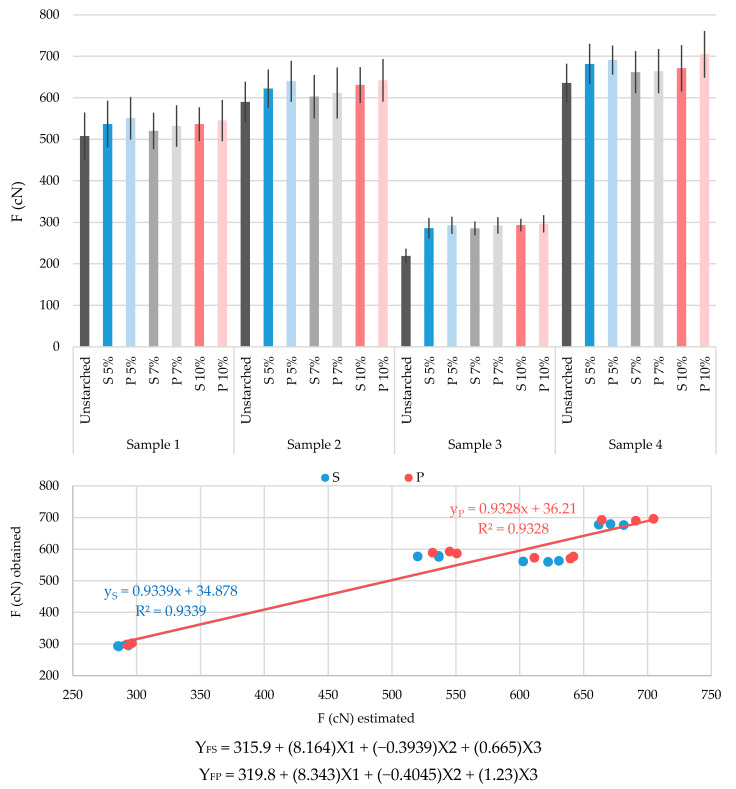
Diagram of breaking forces (F) of tested yarns: unstarched yarn and yarn starched by standard process (S) and by prewetting process (P), with 5%, 7% and 10% starch mass concentration.

**Figure 3:**
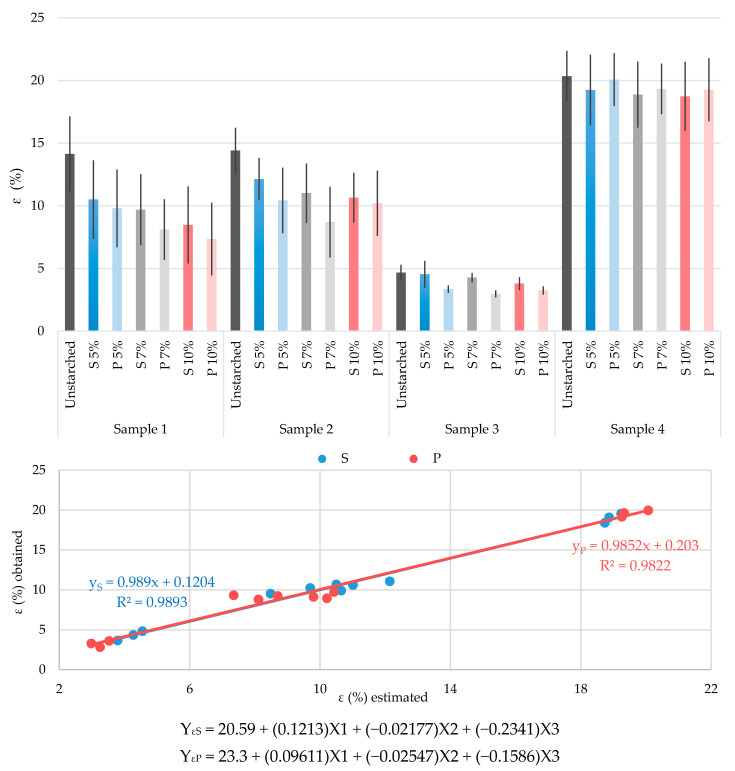
Diagram of elongation at break (ε) of the tested yarns: unstarched yarn and starched yarn by standard and prewetting processes, with 5%, 7% and 10% starch mass concentration.

**Figure 4:**
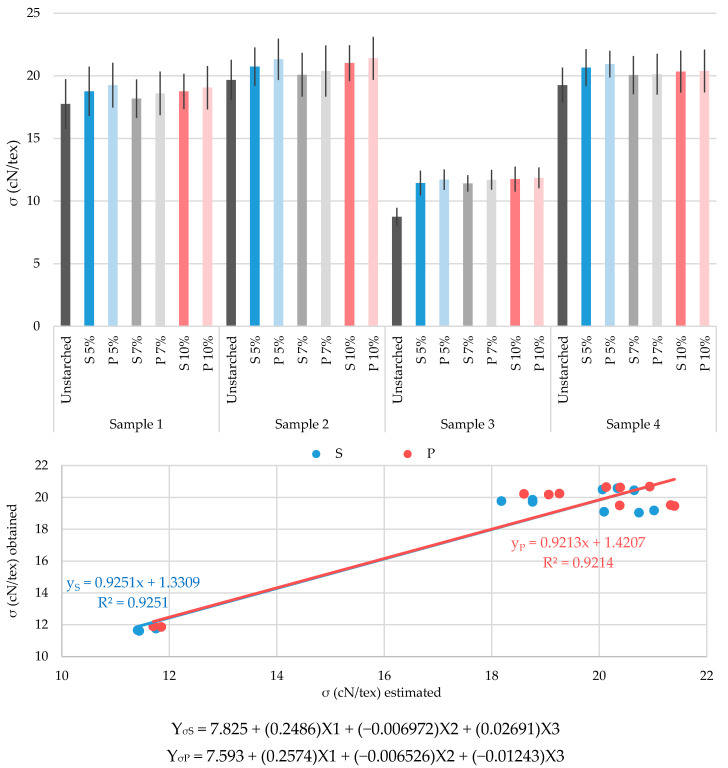
Diagram of tenacity (σ) of the tested yarns: unstarched yarns and yarns starched by standard and prewetting processes, with 5%, 7% and 10% starch mass concentration.

**Figure 5:**
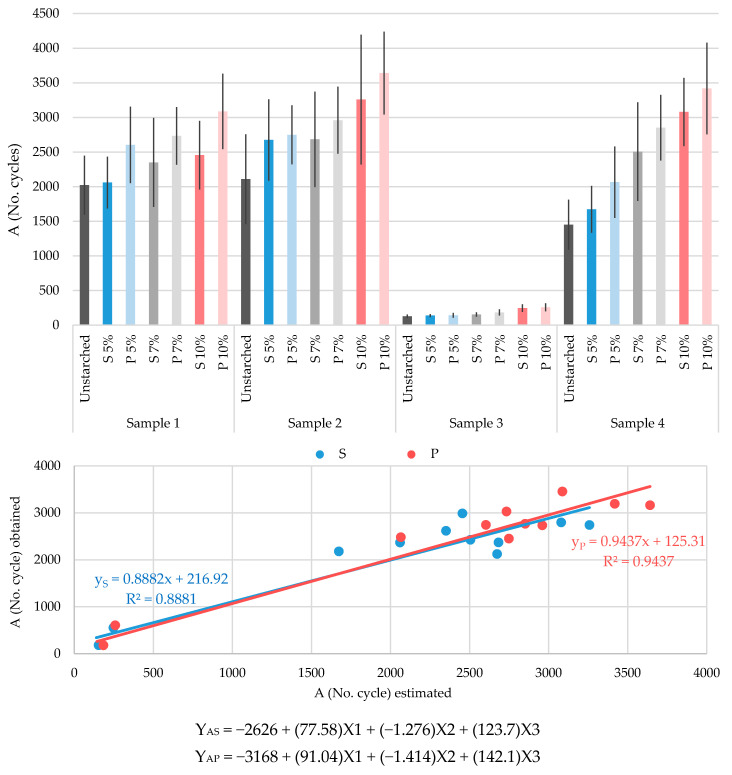
Diagram of abrasion resistance (A) of the tested yarns: unstarched yarns and yarns starched by standard and prewetting processes, with 5%, 7% and 10% starch mass concentration.

The results of conducted tests of yarn breaking properties and abrasion resistance (before and after starching, with and without prewetting and with three starch mass concentrations) are shown graphically in Figure 2, Figure 3, Figure 4 and Figure 5. They are crucial for defining the success of the starching process, and thus continuing the weaving process and for proving the justification of the process described.

The results obtained and their multiple regression analysis show that there is a strong influence of a large number of existing independent variables (predictors) on the dependent variance (criterion) and it is possible to achieve a high correlation. The aim of the regression analysis is to determine the extent to which the linear combination of predictors interprets the variations of the criteria and the extent of the contribution, i.e., the importance of individual predictors. Predictors that are of great importance for achieving maximum correlation with the criterion depend on the criterion itself (YS represent the criterion for the standard starching process, YP represent the criterion for the prewetting starching process). In this case they were yarn real fineness before starching (X1), yarn twists before starching (X2) and starch mass concentration (X3). Graphical representations of multiple regression analysis of the results obtained, with the corresponding equations of multiple regression and highly achieved correlations between the values obtained by testing and the statistically estimated criteria, are shown in Figure 2, Figure 3, Figure 4 and Figure 5.

Figure 2 shows the values of the breaking forces of the tested samples. With starching, the breaking force of all samples increases, which was expected, because breaking force increase is one of the main reasons for implementing the starching process. The smallest breaking force increase is evident for all yarns starched with a mass concentration of 7% by an average of 2–4% for the standard process and 4–5% for the prewetting process, with the exception of sample 3, where this increase is significant and amounts to 30% and 34%, respectively. Opposed to that, observing the results of samples 1, 2 and 4, starched with mass concentrations of 5% and 10% (without significant deviations between the two concentrations), slightly larger improvements of this property are visible (on average 6–7% for the standard process and 8–11% for the prewetting process, while the results of sample 3 remain within the same values). That leads to the conclusion that by increasing the starch mass concentration (and thus starch pickup on the yarn) does not positively affect the increase of breaking force. Based on the results presented, it is easy to conclude that the prewetting starching process gives better results for breaking force increase and the adequacy of the starch mass concentration of 5%. This is extremely important from the aspect of saving starch and other resources. The multiple regression equations are presented separately for the standard starching process and for the prewetting starching process, as well as for the linear regression and for correlation equations between the values obtained by the testing and analysis of the estimated breaking force values.

Tests of elongation at break conducted on starch yarns give very interesting results (Figure 3). In general, yarns starched with a mass concentration of 5% obtain the best results, whereas starched yarns show the smallest decrease in elasticity, which is crucial for the starching process, in order to enable successful formation of the shed with as few as possible interruptions of warp threads. The results are as follows: sample 1—decrease of 25.8% in relation to nonstarched yarns for the standard process and 30.7% in relation to nonstarched yarns for the prewetting starching process (with a difference between the two processes of only 6.7%); sample 2—15.8% in relation to nonstarched yarns for the standard and 27.6% for the prewetting process (with a difference between the two processes of 14.1%); sample 3—2.6% in relation to nonstarched yarns for the standard and 28.3% for the prewetting process (with a difference between the two processes of 26.4%); sample 4—5.4% in relation to nonstarched yarns for the standard and 1.3% for the prewetting process (with a difference between the two processes of only 4.3%). This means that starch from 5% concentration starch mass has the best effect on yarns applying both processes, giving the yarn high strength while retaining elasticity, but the advantage of the prewetting process is obvious.

Increasing the concentration of starch mass reduces the properties of elongation at break. This decrease is quite pronounced for samples 1, 2 and 3 (from 15% to almost 50%), especially by applying the prewetting process. On the other hand, this decrease is almost imperceptible for sample 4 (only 1% to a maximum 8%), where the prewetting starching process is additionally much more suitable. The difference between the two starching processes is most pronounced in sample 3, where the differences reach as much as 30% in favor of the standard starching process.

The correlation coefficients between values obtained by testing and values estimated by the multiple regression analysis, for both starching processes, are extremely high (R^2^ = 0.98).

The results of the tested tenacity property show the expected values, since tenacity is a parameter that puts the property of breaking force and yarn fineness in the ratio (Figure 4). Greater tenacity increase is evident for yarns starched with the prewetting process with all starch mass concentrations, to a greater or lesser extent. What is important to point out once again is that the most significant results of the increase are obtained on yarns starched with the lowest mass concentration (5%): sample 1 at 5.7%, sample 2 at 5.5%, sample 3 at 30.7% and sample 4 at 7.2% by applying the standard starching process; and sample 1 at 7.8%, sample 2 at 8.5%, sample 3 at 34.2% and sample 4 at 8.7% by applying the prewetting starching process. The weakest results of tenacity increase are on yarns starched with 7% mass concentration. Again, sample 3 shows the smallest deviations between the starched yarns, but simultaneously the largest increase in tenacity compared to unstarched yarns.

Multiple regression equations are obtained by analyzing the predictors that affect the tenacity criterion. By linear regression of values obtained by testing and estimated values of tenacity, a high degree of correlation is reached for both starching processes.

Figure 5 shows the abrasion resistance results of the tested unstarched yarns and yarns starched with all three concentrations, using both starching processes. What is most noticeable is that by increasing the starch mass concentration, the abrasion resistance for all tested samples also increases. Sample 3 stands out again, due to significantly lower values compared to other tested yarns. The abrasion resistance of unstarched yarns of samples 1 and 2 does not differ much, only 4%. However, by increasing the starch mass concentration and additionally by applying the prewetting starching process, this difference significantly increases (up to over 50%, or even 70% by applying the prewetting process with a 10% mass concentration). What is difficult to notice in Figure 4, but can be easily read from Table 5, is the fact that starched yarns of sample 3 achieve a marked increase in abrasion resistance, which by increasing the starch mass concentration (with a final value of 10%), reach values of even 91.5% for standard starching and 100.4% for the prewetting starching process. Finally, based on the results obtained it can be concluded that sample 4 records the most significant changes, i.e., increases in abrasion resistance in relation to unstarched yarn, applying all mass concentrations, as well as starching processes. Consequently, the abrasion resistance values increase with increasing starch mass concentration up to as much as 112.2% for standard starching and 135.6% for the prewetting starching process, compared to unstarched yarn.

By comparative analysis of the values of the tested abrasion resistance property, it can be concluded that by increasing the starch mass concentration the abrasion resistance of the tested yarns also increases. The most prominent results were obtained by starching with a mass concentration of 10%, which was not the case with the previously tested properties. In addition, it can be concluded that better resilience is achieved by applying a prewetting process, which also has a number of other advantages over the standard conventional process, such as cost reduction (water, electricity and starch) and the overall environmental aspect.

For the abrasion resistance property, a multiple regression analysis was also performed. Based on the estimated values obtained by linear regression analysis together with the values obtained by testing, the correlation values R^2^ = 0.8881 was achieved for the standard starching process and R^2^ = 0.9437 for the prewetting starching process.

## 4. Conclusions

Based on the results obtained by researching the purpose of starching yarns intended for the production of multifunctional woven fabrics for thermal protective clothing, the following conclusions can be summarized:

Yarn breaking force:

- The increase in breaking force is not accompanied by an increase of starch mass concentration.
- The smallest increase in breaking force is evident by applying a starch mass concentration of 7%.
- Starch mass concentrations of 5% and 10% do not show significant deviations, meaning that it is unnecessary to starch the yarn with a concentration mass above 5%.
- The prewetting starching process gives better results by increasing the breaking force for all tested samples.
- The indicator of the performed analysis of linear regressions and correlations between the estimated values and the values obtained by testing gives a high correlation coefficient (for S: R^2^ = 0.9339 and P: R^2^ = 0.9328).

Elongation at break:

- Yarns starched with mass concentration of 5% show the best results, i.e., the smallest decrease in elasticity.
- Yarns starched by the prewetting process show the smallest elasticity decrease, especially the yarn sample composed of para- and meta-aramid fibers.
- The indicator of the performed analysis of linear regressions and correlations between the estimated values and the values obtained by testing gives a high correlation coefficient (for S: R^2^ = 0.9893 and P: R^2^ = 0.9822).

Tenacity:

- The tenacity increase is more pronounced for yarns starched with the prewetting process with all mass concentrations.
- By applying a mass concentration of 5%, the best results of yarn tenacity are obtained.
- The indicator of the preformed analysis of linear regressions and correlations between the estimated values and the values obtained by testing gives a high correlation coefficient (for S: R^2^ = 0.9251 and P: R^2^ = 0.9214).

Abrasion resistance:

- By increasing the mass concentration, abrasion resistance increases, especially by applying the prewetting starching process.
- The greatest impact of the starching process on abrasion resistance increase is evident for the yarn sample composed of para- and meta-aramid fibers.
- Linear regressions and correlations between testing obtained and analysis of estimated values of wear resistance gives a high correlation coefficient (for S: R^2^ = 0.8881 and P: R^2^ = 0.9237).

It is important to point out that this research gives us an answer to the question: whether yarns used for this specific purpose should be subjected to a starching process and under what conditions. According to the results obtained, it can be finally concluded that the starching process is extremely important in the yarn preparation process. It cannot be neglected and skipped in case of spun yarns of different structures and raw materials. Depending on the yarn composition and its characteristics, individual yarn properties are improved by starching, to a greater or lesser extent. This research proves the justification of high-strength yarn starching, which is reflected in the increase of abrasion resistance, and less in the increase of the breaking force property. The results presented confirm the fact that synthetic and other fibers (which achieve satisfactory yarn strength) still need to be starched, but with lower starch mass concentrations, in order to protect yarn from destruction in the weaving process due to cyclic loads, abrasion on loom elements and static electricity. This research established that the increase of starch mass concentration (and thus starch pickup on yarn) is not accompanied by the improvement of some parameters. In this study, the best results of the tested properties were shown by yarns starched with a lower mass concentration (5%), which indicates the importance of optimizing the starch mass concentration for each type of yarn, implying lower costs, better quality and efficiency in the weaving process, as well as less environmental pollution.

By applying a prewetting starching process, significantly better results were achieved compared to the standard starching process. Starching of different yarn types using two starching process and different starch masses concentrations, as well as their comparison, certainly represents a strong scientific step forward. The application of such knowledge in the textile industry through the implementation of a new starching process, assuming the initial investment, will result in large savings in various aspects of the fabric production process, in this increasingly widespread field of technical textiles.

## Figures and Tables

**Table 1:** Tested yarn properties.

Parameters	Sample I		Sample II		Sample III		Sample IV	
Raw material	PA	cotton	PA	cotton	cotton	MAC	p-AR	m-AR
Ratio (%)	50%	50%	50%	50%	45%	55%	5%	95%
Finesses (Nm)	Nm 70/2		Nm 65/2		Nm 40/1		Nm 60/2	
Real finesses (Nm)	69.36/2		65.55/2		39.69/1		59.28/2	
Twists (twist/m)	787.10		748.16		891.70		323.20	

**Table 2:** Yarn performance characteristics.

m-AR (meta-aramid)	High inherent flammability (high LOI, marginal oxygen index, 27–42) High strength Good dimensional stability Inferior textile-process properties
p-AR (para-aramid)	High inherent flammability (high LOI, limit oxygen index, 29) High strength, dimensional stability and resistance to chemicals Inferior textile-process properties Low degree of stability to solar radiation
MAC (modacrylic)	High inherent flammability (high LOI, marginal oxygen index, 30) High degree of compatibility in the mixture Good fire resistance
PA (polyamide)	Low inherent flammability (low LOI, limiting oxygen index, 20) High strength Antistatic properties Good dimensional stability
Cotton	Flammable (low LOI, oxygen limit, 16–18) High comfort properties Increased wet strength Poor dimensional stability

**Table 3:** Starch agents and their proportions in starch mass.

Starch Mass Composition	Industrial Values		Laboratory Values	
	L or kg	vol (%)	L or kg	vol (%)
Water (L)	500	88.03	5.00	88.03
Fibrostin C75 (kg)	50	8.80	0.50	8.80
Inex 773C (kg)	15	2.64	0.15	2.64
Avilor 308AS (kg)	3	0.53	0.03	0.53
Starching mass (L)	568	100	5.68	100

**Table 4:** Conditions during the starching process.

Condition	Value
Thread tension before prewetting box	40 cN
Water temperature in the prewetting box	65 °C
Starch temperature in the starch box	75 °C
Starching speed	3 m/min
Pressure on the last pair of rollers for squeezing excess starch mass	19.1 N/cm^2^
Temperature on the contact dryer cylinders	140 °C

**Table 5:** Statistical indicators of the tested yarn values.

		Sample 1			Sample 2		
		$\bar{X}$	CV (%)	p_gg_ (%)	$\bar{X}$	CV (%)	p_gg_ (%)
F (cN)	Unstarched	507.62	10.53	2.84	589.92	7.73	2.07
	S 5%	536.64	10.49	2.65	622.17	7.37	1.86
	P 5%	550.76	9.32	2.36	639.75	7.73	1.96
	S 7%	520.05	7.99	2.14	602.68	7.23	2.20
	P 7%	531.84	9.37	2.37	611.43	7.86	2.54
	S 10%	536.54	7.53	1.90	630.60	6.82	1.72
	P 10%	545.13	9.43	2.30	642.05	7.99	2.02
ε (%)	Unstarched	14.15	21.09	5.35	14.41	12.66	3.20
	S 5%	10.50	29.87	7.57	12.14	13.81	3.50
	P 5%	9.80	31.69	8.00	10.43	25.10	6.36
	S 7%	9.70	29.20	7.38	11.01	21.00	5.42
	P 7%	8.11	33.90	7.52	8.70	32.41	8.20
	S 10%	8.48	36.15	9.16	10.65	18.73	4.73
	P 10%	7.35	39.49	9.98	10.21	23.17	6.44
σ (cN/tex)	Unstarched	17.75	10.53	2.84	19.66	7.73	2.07
	S 5%	18.76	10.49	2.66	20.74	7.37	1.87
	P 5%	19.26	9.32	2.35	21.33	7.73	1.96
	S 7%	18.18	7.99	2.14	20.09	7.23	2.20
	P 7%	18.60	9.37	2.37	20.38	7.86	2.53
	S 10%	18.76	7.53	1.90	21.02	6.82	1.72
	P 10%	19.06	9.43	2.30	21.40	7.99	2.02
A (No. cycle)	Unstarched	2021.75	21.05	5.33	2108.30	30.74	7.78
	S 5%	2059.90	18.19	4.60	2674.40	21.93	5.55
	P 5%	2603.00	21.20	5.36	2748.70	15.50	3.92
	S 7%	2350.85	27.29	6.91	2683.10	25.72	6.51
	P 7%	2733.30	15.24	3.86	2960.00	16.33	4.13
	S 10%	2455.00	20.15	5.10	3258.40	28.79	7.29
	P 10%	3086.90	17.64	4.46	3641.20	16.42	4.16
		**Sample 3**			**Sample 4**		
		$\bar{X}$	**CV (%)**	**p_gg_ (%)**	$\bar{X}$	**CV (%)**	**p_gg_ (%)**
F (cN)	Unstarched	218.77	8.06	2.04	635.44	7.30	1.85
	S 5%	285.99	8.57	2.17	681.38	7.16	1.81
	P 5%	292.77	7.02	1.78	690.88	5.05	1.28
	S 7%	285.34	5.57	1.44	661.83	7.61	1.93
	P 7%	292.52	6.94	1.71	664.21	8.06	2.03
	S 10%	293.52	8.44	1.29	671.12	8.29	2.10
	P 10%	296.35	7.04	1.76	704.73	7.99	2.02
ε (%)	Unstarched	4.67	13.25	3.36	20.34	9.97	2.53
	S 5%	4.55	23.44	5.95	19.24	14.68	3.71
	P 5%	3.35	9.22	2.34	20.07	10.50	2.66
	S 7%	4.27	8.55	2.19	18.87	13.91	3.53
	P 7%	2.98	9.25	2.38	19.33	10.38	2.63
	S 10%	3.79	13.58	3.40	18.74	14.70	3.71
	P 10%	3.25	10.28	2.57	19.26	13.09	3.31
σ (cN/tex)	Unstarched	8.75	8.06	2.05	19.26	7.30	1.84
	S 5%	11.44	8.57	2.17	20.65	7.16	1.81
	P 5%	11.71	7.02	1.77	20.94	5.05	1.28
	S 7%	11.41	5.57	1.44	20.06	7.61	1.93
	P 7%	11.70	6.94	1.71	20.13	8.06	2.04
	S 10%	11.75	8.44	2.15	20.34	8.29	2.09
	P 10%	11.85	7.04	1.77	20.39	7.99	2.12
A (No. cycle)	Unstarched	129.10	20.28	5.13	1450.85	24.81	6.28
	S 5%	138.85	15.26	3.86	1673.50	20.27	5.13
	P 5%	143.15	24.29	6.15	2065.20	24.97	6.32
	S 7%	153.70	21.02	5.32	2505.50	28.42	7.19
	P 7%	184.05	24.83	6.28	2852.00	16.57	4.19
	S 10%	247.20	22.04	5.58	3079.25	15.97	4.04
	P 10%	258.70	22.63	5.73	3418.60	19.32	4.89

where F (cN)—breaking force, ε (%)—elongation at break, σ (cN/tex)—tenacity, A (No. cycles)—abrasion resistance, $\bar{X}$—mean, CV (%)—coefficient of variation, p_gg_ (%)—practical error limit; S 5%—standard starching process with 5% starch mass concentration, P 5%—prewetting starching process with 5% starch mass concentration, S 7%—standard starching process with 7% starch mass concentration, P 7%—prewetting starching process with 7% starch mass concentration, S 10%—standard starching process with 10% starch mass concentration, P 10%—prewetting starching process with 10% starch mass concentration.

## Data Availability

The data presented in this study is openly available.
